# Blockchain in radiology research and clinical practice: current trends and future directions

**DOI:** 10.1007/s11547-022-01460-1

**Published:** 2022-02-23

**Authors:** Alberto Stefano Tagliafico, Cristina Campi, Bignotti Bianca, Chandra Bortolotto, Duccio Buccicardi, Coppola Francesca, Roberto Prost, Marco Rengo, Lorenzo Faggioni

**Affiliations:** 1 IRCCS Ospedale Policlinico San Martino, Genova, Genoa, Italy; 2 Department of Health Sciences (DISSAL), University of Genoa, Genoa, Italy; 3 Dipartimento Di Matematica, Università Di Genova, via Dodecaneso 35, 16146 Genova, Italy; 4 Dipartimento Di Radiologia, Fondazione IRCCS Policlinico San Matteo, Pavia, Italy; 5 S.C. Radiologia P.O. Levante, ASL2 Savonese, Savona, Italy; 6 Department of Radiology, IRCCS Azienda Ospedaliero-Universitaria Di Bologna, Bologna, Italy; 7 Azienda Ospedaliera Brotzu, Cagliari, Sardegna Italy; 8 Department of Radiological Sciences, Oncology and Pathology, Sapienza University of Rome - I.C.O.T. Hospital, Via Franco Faggiana, 1668, 04100 Latina, Italy; 9 Diagnostic and Interventional Radiology, University Hospital of Pisa, Via Paradisa 2, 56100 Pisa, Italy

**Keywords:** Blockchain, Radiology, Imaging, Informatics, Technology

## Abstract

Blockchain usage in healthcare, in radiology, in particular, is at its very early infancy. Only a few research applications have been tested, however, blockchain technology is widely known outside healthcare and widely adopted, especially in Finance, since 2009 at least. Learning by history, radiology is a potential ideal scenario to apply this technology. Blockchain could have the potential to increase radiological data value in both clinical and research settings for the patient digital record, radiological reports, privacy control, quantitative image analysis, cybersecurity, radiomics and artificial intelligence.

Up-to-date experiences using blockchain in radiology are still limited, but radiologists should be aware of the emergence of this technology and follow its next developments. We present here the potentials of some applications of blockchain in radiology.

## Introduction

During Covid-19 pandemic, perhaps due to the lockdown periods, the term blockchain received a boost of popularity. Blockchain is related to the first cryptocurrency, Bitcoin, first described in the Satoshi Nakamoto white paper who proposed an innovative method for electronic transactions without relying on trust using digital signatures in a peer-to-peer network using proof-of-work to record a public history of transactions [1, 2]. Although blockchain technology was first described in a 1991 article delineating ways to certify and timestamp digital data [1, 2], only in this period blockchain can be considered a popular topic in Finance. Furthermore, blockchain is slowly but progressively entering other sectors such as healthcare [3–7]. However, despite blockchain's potential to create healthcare-related data even more secure and transparent than current technology, the majority of radiologists are largely unfamiliar with the potential of this disruptive technology. Only a few papers have been published so far in radiological literature regarding blockchain, albeit this technology is considered by the European Union a potential breakthrough technology for decades to come to control and share access to data in a secure, transparent, certifiable way without intermediaries [8]. The purpose of this article is to give a simple and practical overview for the general radiologists of potential applications and implications of this technology in the radiological environment. Awareness of this topic is up-to-date especially for younger radiologists who will probably face this technological innovation.

### What is Blockchain?

**Fig. 1:**
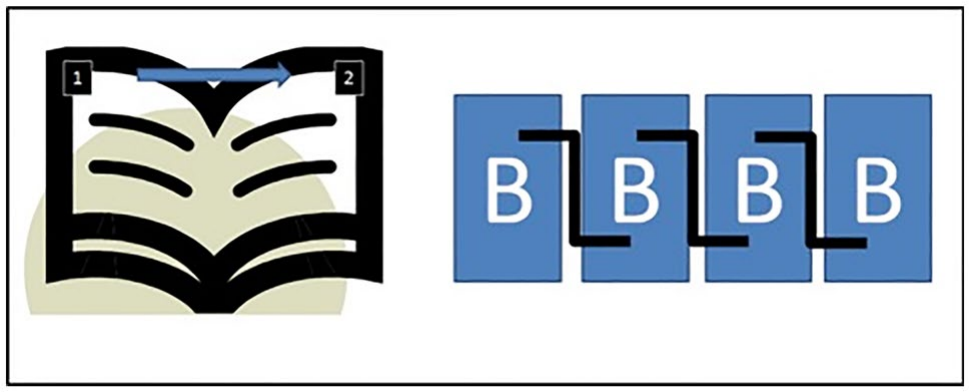
Schematic drawing showing blockchian principle

Blockchain can be simply described as a distributed digital ledger. The digital ledger is used to keep a record of every activity made by many, and the ledger is shared and decentralized, therefore there is not a single centralized point of control. In other technical words, blockchain can be considered a “cryptographically secure transactional singleton machine with shared-state” [9]. Complex mathematical algorithms that are more than extremely hard to break, are driven by a single global truth generated by a computer that everyone believes in and is open to everyone. To simplify for general radiologists not keen on complex computer applications, a blockchain could be considered a chronological chain of “blocks” where each of them contains both and unique hash (a hash is a long alphanumeric string created using an algorithm that serves as a fingerprint, or unique identifier, of digital data [10]). Data of the sender and the receiver contain the data to be considered and a hash with the data related to the content of the previous and current block. Linking the hashes creates a chain, a chain of “blocks” adding several layers of security to the data. Every computer belonging to the distributed, peer-to-peer network has a ledger which means a bookkeeping system details of individual transactions. A copy of the public ledger working through consensus has to be created by every computer. To alter this chain, replication of more than 50% of the network would have to be done for a new block accepted. Therefore, as long as the network enlarges, it becomes virtually impossible to tamper. Blockchain is considered to be “immutable” and secure for this reason. Online resources are available to acquire deeper knowledge of blockchain details which are outside the purpose of this review [3–6, 8, 10–15]. In financial activities, one of the goals of blockchain was to by-pass bank and regulatory issues. Indeed, blockchain technology appeared to a broad community in 2008 during the economic financial crisis [16]. In 2008, at the end of the financial crisis, a still unknown group of people named under the name “Satoshi Nakamoto” proposed through a white paper a public peer-to-peer monetary system, available to the general public, which has a popular name in 2021: “Bitcoin” [17–19]. In Bitcoin, the blockchain protocol allows sending payments online without banks or other third parties. After Bitcoin, a researcher and computer operator named Vitalik Buterin created and made publicly available the Ethereum network. The Etherum network is another public peer-to-peer blockchain where users can insert self-executing transactions within computer code verified and executed automatically in a trustless manner [4, 17–19]. This method can be considered a smart contract and has several functions. In addition, developers around the world have access to the code for both Bitcoin and Ethereum, and the technology can be used to create smart contract and verification functions [15]. At present, blockchain technology has not been tampered or hacked, there has been no possibility to hack this technology, and the value of “bitcoin” and other projects related to blockchain-based cryptocurrencies have risen a lot since 2008 confirming the growing interest in this technology so far. Several blockchain-related activities do not need to create a new blockchain network ex-novo to create smart contracts, but can rely, for example, on Ethereum network [15]. Simplifying a lot, a blockchain is just a file creating connections with the other blocks like page numbers in a book, then blocks in a chain refer to previous blocks, like page numbers links previous and following pages in a book (Fig. 1). A literature search on PubMed and Embase with relevant studies regarding blockchain and medical imaging (search strategy: “blockchain and medical imaging” performed on 20 November 2021) is reported on Table 1 [3–7, 12–14, 20–27]. Several papers in the literature advocate the possible future usage of blockchain in different healthcare environment, including radiology. Data security, including radiological data, is one of the most important filed where blockchain is foreseen to be adopted. However, at the present time, radiological literature and scientific community is waiting for new original articles testing the potential applications of blockchain in a clinical environment. An increasing trend of papers published in medical literature is demonstrated by 3 papers in 2018 and 27 in 2021 (search strategy: blockchain and imaging). There is clearly a growing awareness and interest on this topic.

**Table 1:** This table reports literature search on PubMed and Embase with all relevant studies regarding blockchain and medical imaging

Author	Type of Publication	Scientific Category	Year of Publication	Journal	Aim
McBee MP [20]	Review	Radiology	2020	J Digit Imaging	Principles
Patel V [22]	Review	Health Policy and Services	2019	Health Informatics	Principles
European Society of Radiology (ESR) [28]	Review	Radiology	2021	Insights Imaging	Basic technology and terminology
Pilozzi A [21]	Review	Neurology	2020	Brain Sci	Data security
Glicksberg BS [14]	Original Research	Internal Medicine	2020	J Med Internet Res	Data security
Abdullah S [12]	Review	Radiology	2020	Acad Radiol	Basic
Leeming G [23]	Review	Internal Medicine	2019	Front Med (Lausanne)	Data security
Kumar R [27]	Original Research	Computer Science	2020	Comput Med Imaging Graph	Data security
Cunningham J [26]	Review	Health Policy and Services	2017	Stud Health Technol Inform	Data security
Sultana M [32]	Original Research	Computer Science	2020	BMC Med Inform Decis Mak	Data security
Verde F [24]	Review	Radiology	2019	Journal of digital imaging	Basic Usage

Search strategy: “blockchain and medical imaging” performed on November 2021

### Blockchain in radiology

Radiologists are used to deal with technological challenges because, since the beginnings of its history, radiology has been the playfield of technological development.

Young radiologists should be aware in the near future of the basic principles of blockchain, of the potential of this technology and its limitations. Radiologists do not need to know the deepest details of blockchain, but general awareness of blockchain potentiality is necessary especially with the rise of artificial intelligence in radiology [12–14, 20, 24, 25, 28].

### Potential applications of blockchain in radiological clinical practice

There are several potential applications of blockchain in clinical practice:

- Patient digital record

**Fig. 2:**
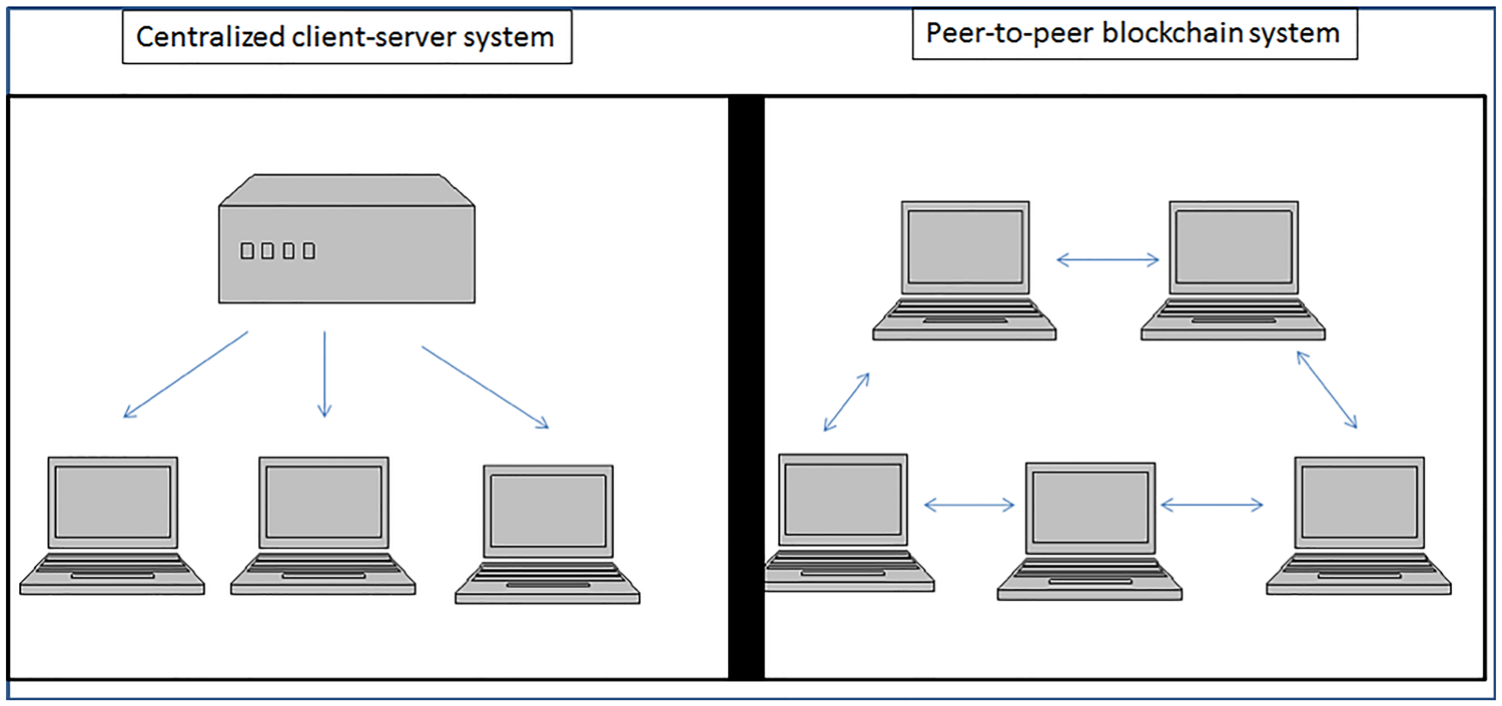
Peer-to-peer blockchain system versus centralized client server system

Up-to-date radiological images are stored in hospitals or clouds using central databases where images are then, on-demand, transferred to physical media such as DVD or hard-disks for example. This standard process has several limitations such as obvious time constraints if there is an urgent need to have copies of centrally stored images and if there is the possibility of data damage and loss if the central storage site is corrupted-up and fails. In 2018, it has been developed a framework using blockchain technology to permit patients to allow electronic access to their medical imaging data preserving security [22]. The goal of blockchain usage was to set a series of predetermined endpoints to retrieve imaging studies and patient data ensuring that only authorized entities could have access only to the patient's desired data [22]. In other word, patients can easily access their electronically stored health information and share part of it at their discretion without the need of a central authority (e.g., The hospital database) because the patient is a node of the chain (Fig. 2).

This context permits protected and decentralized distribution and sharing of medical data. The patient, who is the owner of his data as guaranteed by law, practically own his images data and can choose how and when healthcare professionals can access these data. In the blockchain process, every single action related to image usage can be tracked as a list of users with record and immutable signs related to the permission to access each single study. Up-to-date patients are used to signing an informed consent (on paper or paperless using electronic data sets) to allow usage of their radiological images, and this permission can be withdrawn at any moment, however, practically, it could be difficult to have timely access to these records.

Finally, using blockchain, it would be possible to have an immutable track record of radiation exposure related to every single procedure.

- Radiological report

It is widely accepted that second-look evaluation of radiological images can strongly enhance Radiologist performance, especially in sub-specialized areas such as musculoskeletal, neuro and breast radiology [29, 30]. In these cases, there is often a non-synchronous evaluation made by different specialists such as combination of nuclear medicine physicians/radiologist reporting on PET/MR or second reader of breast screening procedure and re-evaluation of complex imaging studies by sub-specialized radiologists. Blockchain is an opportunity to clearly separate and keep an immutable track record of every single input to the imaging study under investigation. In this way, the contribution of every professional is recorded and acknowledged, providing the extent of every single contribution and even the responsibility. In future, blockchain would help in differentiating human (the radiologist), contribution to the diagnosis and the contribution of AI tools. The possibility to separate human from AI contribution could have practical implications for ethical and medico-legal issues [31].

Finally, digital data in PACS and RIS may require correction and modification due to several errors independent from the Radiologist actions such as anagraphical errors at the time of registration. These errors are often corrected after the final report signature and could pose medico-legal issues. The use of blockchain could be very useful to track these changes and to avoid any misinterpretation of data modifications after electronic report signature. An example of a proposed platform that enhances the security of medical records and images transmission through a combination of blockchain and zero trust principles was proposed in 2020 by Sultana et al. [32]. The authors found that blockchain technology ensured data integrity by recording every transaction with effective encryption preventing data vulnerabilities. Adopting this technology has several advantages, as discussed so far, however, several disadvantages have to be considered. One is the network speed due to the need to have peer-to-peer verification using a public blockchain with several nodes. The other disadvantage is the high energy usage for node performance. Finally, it is important to have a correct key management to avoid key loss which would be difficult to overcome.

### Potential applications of blockchain in radiological research

Several research activities can be enhanced by the application of blockchain. The following list describes the major applications of this technology in radiological research, but it is likely that in future a lot of new applications will be found.

- Data entry: patient data and privacy control
- Quantitative image analysis
- Reader verification: co-workers authentication
- Cybersecurity
- Radiomics and artificial intelligence

Regarding patient data and privacy control, significant data related to the research project under investigation can be stored securely and with an immutable track-record guaranteed by blockchain technology. The patient can directly insert at his discretion only relevant information for the current research project, for example if the project is related to MRI or CT the patient can insert information regarding the presence of metallic hardware or allergy to iodinated contrast media. These data will be available to all institutions participating in the project without disclosing every data of an electronic health record that may contain a huge amount of data, the majority not relevant to the radiological research under investigation. Using blockchain patient data and privacy on the single research project will be transferred among researchers and institution safely without risking data leak and allowing the patient to withdraw consent or data at any time without the need to fill out new paper-based consent forms which are difficult to be stored and retrieved. In this case, maximum compliance with all regulatory issues is largely guaranteed sparing time and physical resources to store documents across institutions. Databases for research purposes built using blockchain will be different from those created traditionally: those differences can be found in Table 2. The key-point is that using blockchain the process of database creation does not need trust because data entry and subsequent modification are decentralized, completely traceable and do not have the possibility to be deleted. In other words, a database built on a blockchain system contains a standard database with the addition of some software that adds new rows added and validated according to predefined rules and receives new rows to its peers across a network where all peers possess the same data simultaneously (Table 2).

**Table 2:** Standard database vs. Blockchain-based database

Asset	Standard Database	Database using Blockchain
Consistency of data	✔✔✔	✔✔✔
Security of Data	✔	✔✔✔
Integrity of Data	✔	✔✔✔

In quantitative data analysis, it is important to guarantee repeatability of research methods, which has been, and it is still a critical issue in radiomics and artificial intelligence applications [33–36]. With blockchain, it would be possible to have a clear and immutable track record of every step to generate the data for AI algorithms. Indeed, by the majority of radiologists it is difficult or almost impossible to understand exactly how radiomics and AI data come from and how image selection and analysis have been done. In addition, data sharing of radiological research is still in its infancy and privacy, and regulatory issues are a huge obstacle to repeatability assessment of radiomics and AI-based radiological research. As a consequence, the process of clinical implementation of radiomics and AI application in clinical practice is slow. Since the first definition of radiomics in 2012 [37], no clinical application of this technology is still available for wide usage in clinical practice. Blockchain applications have the possibility to exactly track every step of radiomics and AI workflow to ensure repeatability assessment of results with an obvious spare of time and money. In addition, the use of blockchain can be used to track the readers or investigators involved in research activity and to authenticate unequivocally co-workers avoiding or at least complicating and discouraging typical research misconduct such as ghost authorship for guest authorship [38–40]. Furthermore, it would be possible to find records of every process of quantitative image evaluation such as ROI positioning, image selection, etc., to assess where and how a process of the research under investigation influenced the final data. Blockchain is suitable for tracking annotations on medical images made by different readers and to approve the changes or the opinions only if consensus is reached over a determined cut-off and penalizing the weight of the opinions or judgment if consensus is weak. In this scenario, blockchain could be considered a kind of advanced Delphi process. Finally, no data manipulation would be possible because every process has a record in the ledger. Regarding cybersecurity, it is already history that radiological images could be altered by cyber-attacks. For example, adversarial attacks on medical imaging can fool AI systems determining the misclassification of images or even altering images if they are shared through the web [41]. Indeed, if we only change a few pixels in a way that the radiological image looks the same to the radiologist’s eye, the AI tools can fail. In this way, we will partially lose faith in new technological achievements. In this scenario, blockchain can enhance security; a kind of “radiological cybersecurity”, in radiological images usage on-site and on teleradiology facilities by excluding or signaling that images have been altered by non-authorized entities. Indeed, hackers could introduce noise or other modifications to standard images, even not visible to humans, but able to influence AI systems [42]. Critical human, in this case radiological, supervision is necessary to check these processes and to really take advantage in the long term of technology.

In radiomics and AI which are promising technological achievements but not yet established in clinical practice, there is the well-known challenge of repeatability of results due to difficulties in sharing very large databases with clinical, radiological and radiomics data [33]. Blockchain could offer an opportunity to avoid manipulation of data by assuring and tracking every access and modification to the original database by personnel authorized or ever by patients who can easily and efficiently retract consent at any time.

An innovative research study investigating the implementation of various deep learning models over the blockchain to improve lung cancer detection was done by Kumar et al. [27]. In addition, the authors proposed a unique method combining locally learned deep learning models over the blockchain to improve lung cancer evaluation [27].

### Difficulties in practical blockchain application

There are several difficulties in the application of blockchain in a healthcare environment. One of the first is the limited experience of the final user in using and managing the key to access the nodes. Technological improvements will be likely to create user-friendly interfaces linked to electronic devices such as smartphones linking biometrical recognition features with cryptographic actions needed to access the decentralized system as a node. In other words, if patients affected by several pathological conditions determine difficulties in storing and retrieving blockchain-related passwords; Governments and Central Institution should work to assure an effective way to take advantage of this technology. Another drawback is that decentralization is quite expensive and energy consuming: the more computers running a code, the more expensive the process. In healthcare, it would be cheaper to centrally store and compute a platform where patients and professionals could log in with their smart contracts after the counterparty signs off, to have both parties rely on the result. Of course, in this way the guarantees of decentralization are lost with the advantages of data quality using blockchain at a very lower price. Blockchain can be public or private. Public vs private blockchain. Intuitively, using a public blockchain anyone is free have the access, read and write access for anyone, the authority is decentralized, however the transition speed is slow, and the efficiency is low. Public blockchain is fully immutable. Conversely in a private blockchain, a single organization has access and authority on the network representing a partially decentralized system. A private blockchain as a fast transaction speed and a high efficiency and in is partially immutable because the operator can override, edit or delete the entries. These characteristics of public and private blockchain have to be considered when deciding to use public or private blockchain.

### Job opportunities in radiological departments

Finally, thanks to the advent of radiomics and AI applications in radiology and the possibility in the near future to take advantage of blockchain, there could be the opportunity for Radiologists and Radiologic department to work with non-medical professionals dedicated to AI, blockchain and even cybersecurity. Radiology has always been at the edge of technological development, and blockchain offers an opportunity to further enhance the value of radiological data and professionalism in the upcoming AI era [35, 43, 44].

## Conclusion

Blockchain technology utilization in healthcare and in radiology is at its very early infancy. Only a few research applications have been tested, however, blockchain technology is widely known outside healthcare and widely adopted since 2009 at least. Given the implementations of new technology such as radiomics and artificial intelligence, radiology is a potential ideal scenario to apply this technology. Blockchain could have the potential to increase Radiological data value in both clinical and research settings. Up-to-date experiences using blockchain in radiology are still limited, but further research is worthy of being explored. Radiologists should be aware of the emergence of this groundbreaking technology and follow its next developments.
